# Time dependent degeneration of the nigrostriatal tract in mice with 6-OHDA lesioned medial forebrain bundle and the effect of activin A on l-Dopa induced dyskinesia

**DOI:** 10.1186/s12868-019-0487-7

**Published:** 2019-02-13

**Authors:** Peggy Rentsch, Sandy Stayte, Gary P. Morris, Bryce Vissel

**Affiliations:** 1 Faculty of Medicine, University of New South Wales, High Street, Sydney, NSW 2052 Australia; 2 Centre for Neuroscience and Regenerative Medicine, Faculty of Science, University of Technology Sydney, PO Box 123, Broadway, Sydney, NSW 2007 Australia; 3 St. Vincent’s Centre for Applied Medical Research (AMR), 405 Liverpool St, Sydney, NSW 2010 Australia

**Keywords:** Abnormal involuntary movements, Neuroinflammation, Parkinson’s disease, Stereology, Striatum, Substantia nigra pars compacta

## Abstract

**Background:**

Accurately assessing promising therapeutic interventions for human diseases depends, in part, on the reproducibility of preclinical disease models. With the development of transgenic mice, the rapid adaptation of a 6-OHDA mouse model of Parkinson’s disease that was originally described for the use in rats has come with a lack of a comprehensive characterization of lesion progression. In this study we therefore first characterised the time course of neurodegeneration in the substantia nigra pars compacta and striatum over a 4 week period following 6-OHDA injection into the medial forebrain bundle of mice. We then utilised the model to assess the anti-dyskinetic efficacy of recombinant activin A, a putative neuroprotectant and anti-inflammatory that is endogenously upregulated during the course of Parkinson’s disease.

**Results:**

We found that degeneration of fibers in the striatum was fully established within 1 week following 6-OHDA administration, but that the loss of neurons continued to progress over time, becoming fully established 3 weeks after the 6-OHDA injection. In assessing the anti-dyskinetic efficacy of activin A using this model we found that treatment with activin A did not significantly reduce the severity, or delay the time-of-onset, of dyskinesia.

**Conclusion:**

First, the current study concludes that a 3 week duration is required to establish a complete lesion of the nigrostriatal tract following 6-OHDA injection into the medial forebrain bundle of mice. Second, we found that activin A was not anti-dyskinetic in this model.

**Electronic supplementary material:**

The online version of this article (10.1186/s12868-019-0487-7) contains supplementary material, which is available to authorized users.

## Background

Parkinson’s disease (PD) is a progressive neurodegenerative disorder characterized by the loss of dopaminergic neurons in the substantia nigra pars compacta (SNpc), leading to a reduction in dopamine availability in the striatum. Clinically, this manifests as motor dysfunction, including tremors, rigidity and bradykinesia [1]. l-Dopa treatment still remains the most effective available therapy to improve these motor symptoms, however long-term use leads to the development of debilitating l-Dopa-induced dyskinesia’s (LIDs) [2]. At present there are few available treatments to reduce the course of LID development.

Several toxin-based animal models of PD are available to investigate the mechanisms of LID development and possible strategies to combat it. The MPTP-lesioned non-human primate [3, 4] and the 6-OHDA lesioned rat [5, 6] are traditionally the most prominent. Although there are clear benefits to establishing mouse models, particularly the ease at which genetically modified lines can now be developed, initial attempts to establish dyskinesia in mice suffered several technical setbacks. In particular, MPTP lesioned mice simultaneously exhibited inconsistent dopamine depletion and required large doses of l-Dopa to develop any dyskinetic behaviours [7, 8], while a LID mouse model with a 6-OHDA lesion resulted in mortality rates of up to 82% [9]. Improvement in mortality rates is observed in the 6-OHDA mouse model when the injection location is shifted from the medial forebrain bundle (MFB) to either intrastriatal or intranigral, but this comes at the cost of reduced and more variable LID expression [10]. More recently, these issues have been overcome by injecting a smaller volume of more concentrated 6-OHDA into the MFB of mice, which has led to reduced mortality rates and more consistent lesions with animals expressing stable LIDs [11]. While the behavioural outcomes have been characterized in detail, the time course of neurodegeneration in this updated MFB 6-OHDA mouse model has gained less attention. Therefore, we first aimed to investigate the progression of neuron loss in the SNpc and terminal loss in the striatum over a 4 week period.

Using this model, our next aim was to then investigate a novel pharmacological approach to prevent, reverse or halt the development of LIDs. It is by now acknowledged that chronic neuroinflammation may play a role in the development of PD pathology [12, 13]. It is also well-established that mouse models of PD, including the 6-OHDA MFB mouse model, recapitulate this phenotype [14]. Conceivably, as recently suggested, neuroinflammation in PD may also be mechanistically linked to the development of LIDs [15]. One attractive hypothesis, for example, suggests that extended l-Dopa therapy may exacerbate the preexisting pro-inflammatory milieu, thereby promoting further neuron loss by shifting glial function more towards a damaging, rather than supportive role, culminating in the development and expression of LID [16, 17]. Support for this idea comes from the prior success of anti-inflammatory therapies, such as corticosterone [18] or nitric oxide synthase inhibitor [19], in reducing the development of LID in rat models of PD. Collectively, these studies indicate further exploration of anti-inflammatories therapies for PD patients with LID is warranted.

Our group has prior experience examining the therapeutic efficacy of putative anti-inflammatories in neurodegenerative disease. In particular, we have previously illustrated that exogenous administration of activin A in the CNS following an acute neurodegenerative injury in mice resulted in reduction in microglial numbers, reduced microglial activation, reduced pro-inflammatory cytokine release and reduced astrogliosis [20]. More recently we found a significant neuroprotective effect of recombinant activin A treatment in the unilateral 6-OHDA and acute MPTP mouse models of PD [21, 22]. Furthermore, we illustrated that activin A can reduce the number of microglia and astrocytes in the SNpc, which were previously elevated through MPTP or LPS toxicity [21], suggesting the mechanism underlying it’s promising effects may be anti-inflammatory. Intriguingly, a recent study illustrated an increase in pro-inflammatory cytokine gene expression, as well as a concomitant up-regulation of anti-inflammatory markers, including activin A, in the striatum of mice with a 6-OHDA MFB lesion [14]. This finding, combined with our prior success with activin A in mouse models of PD, illustrates activin A may be a promising therapeutic for PD, possibly through anti-inflammatory mechanisms. Considering the putative mechanistic link between LID development and neuroinflammation in PD it is possible this therapeutic promise may therefore also extend to LID.

In the following study we therefore first characterized the temporal evolution of neurodegeneration and mortality in the 6-OHDA MFB mouse model of PD. Using this model we then investigated the therapeutic efficacy of activin A to prevent, reverse or halt the development of l-Dopa induced LIDs.

## Results

The 6-OHDA rodent model of PD is a preclinical model currently used to identify promising new therapies for PD and related side effects, such as LIDs. In order to establish the progression of lesion development, mice were unilaterally injected with 6-OHDA or ascorbic acid (vehicle control) into the MFB and nigrostriatal degeneration was assessed over 4 weeks.

### 6-OHDA injection induces cell loss in the SNpc over time

**Fig. 1:**
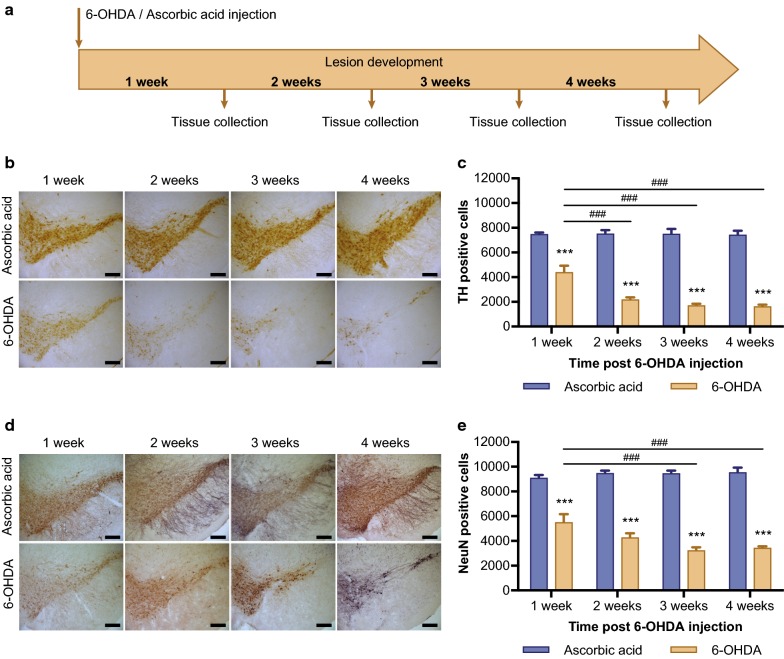
Time dependent loss of TH- and NeuN-positive cell populations in the SNpc following 6-OHDA, **a** experimental design. Mice receive 6-OHDA or ascorbic acid (vehicle control) injection. Tissue was collected after 1, 2, 3 and 4 weeks in order to assess lesion development over time. Representative images of TH (**b**) and NeuN (**d**) positive neurons in the SNpc over time. Stereological quantification of the ipsilateral SNpc resulted in significant and progressive loss of TH (**c**) and NeuN (**e**) positive neuron counts following unilateral 6-OHDA injection. All values represent the mean ± standard error of the mean (SEM). *** = *p *< 0.001 compared to ascorbic acid, ^###^ = *p *< 0.001 compared to 1 week 6-OHDA. (n = 5 per group). Scale bar represents 200 μm

In this experiment there was a 20% mortality rate in 6-OHDA lesioned animals, with 5 of the 25 animals entered into the study dying prematurely. Animals that underwent sham surgery had no lasting post-operative complications. Tissue was collected 1, 2, 3, and 4 weeks post 6-OHDA injection (Fig. 1a) and dopaminergic and total neuron numbers were quantified using stereological analysis of TH and NeuN positive cells, respectively, to assess SNpc dopaminergic neurodegeneration over time. Two-way ANOVA demonstrated a significant interaction between toxin and time on TH positive cell numbers in the SNpc (F_(3,32)_ = 10.31, *p *< 0.001, n = 5 per group) indicating a significant effect of 6-OHDA on dopaminergic cell survival over time. The simple main effect of toxin revealed significant loss of TH positive cell numbers at 1 (F_(1,32)_ = 56.93, *p *< 0.001), 2 (F_(1,32)_ = 171.40, *p *< 0.001), 3 (F_(1,32)_ = 203.49, *p *< 0.001) and 4 weeks (F_(1,32)_ = 203.35, *p *< 0.001) following unilateral 6-OHDA lesion compared to ascorbic acid controls (Fig. 1b, c). The simple main effect of time after 6-OHDA treatment indicated significant loss of TH positive cells with time (F_(3,32)_ = 20.55, *p *< 0.001). A Bonferroni post hoc analysis revealed a significant difference in TH positive cells between 1 and 2 weeks (*p *< 0.001), 1 and 3 weeks (*p *< 0.001) and 1 and 4 weeks post lesioning (*p *< 0.001).

Two-way ANOVA demonstrated a significant interaction between toxin and time on NeuN positive cell numbers in the SNpc (F_(3,32)_ = 7.02, *p *< 0.001, n = 5 per group) indicating a significant effect of 6-OHDA on neuronal cell survival over time. The simple main effect of toxin showed significant loss of NeuN positive cell numbers at 1 (F_(1,32)_ = 61.36, *p *< 0.001), 2 (F_(1,32)_ = 128.82, *p *< 0.001), 3 (F_(1,32)_ = 184.03, *p *< 0.001) and 4 weeks (F_(1,32)_ = 177.59, *p *< 0.001) following unilateral 6-OHDA lesion compared to ascorbic acid controls (Fig. 1d, e). The simple main effect of time after 6-OHDA treatment indicated significant loss of NeuN positive cells with time (F_(3,32)_ = 10.16, *p *< 0.001). A Bonferroni post hoc analysis revealed a significant difference in TH positive cells between 1 and 3 weeks (*p *< 0.001) and 1 and 4 weeks post lesioning (*p *< 0.001).

### 6-OHDA injection induces early terminal loss in striatum

A loss of dopaminergic neurons in the SNpc has been shown to result in degeneration in the striatum of the projecting fibres from the nigral cell bodies [23], subsequently leading to a dopamine deficit and motor symptoms. DAT plays an important role for maintaining sufficient dopamine levels for release into the synaptic cleft in the striatum, and its expression has been shown to reduce following dopaminergic neuron loss in the SNpc [24, 25]. Thus, it is a useful indicator for lesion in the striatum following 6-OHDA. In order to confirm the extent of the lesion to the striatum we quantified TH and DAT expression in the striatum via densitometry at 1, 2, 3, and 4 weeks post 6-OHDA administration.

**Fig. 2:**
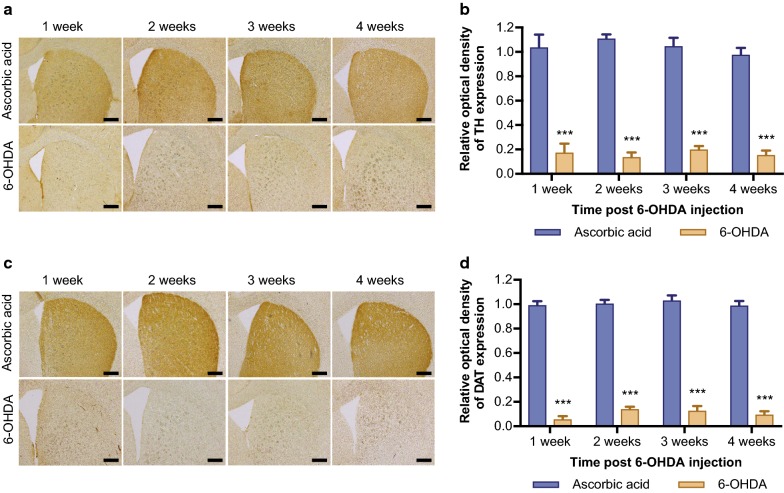
Loss of striatal terminals following 6-OHDA, Representative images of TH (**a**) and DAT (**c**) immunoreactive fibres in the striatum. Optical density analysis in the ipsilateral striatum resulted in significant loss of TH (**b**) and DAT (**d**) expression after 6-OHDA injection that was fully established 1 week after 6-OHDA injection and didn’t progress further over time. All values represent the mean ± SEM. *** = *p *< 0.001 compared to ascorbic acid. (n = 5 per group). Scale bar represents 400 μm

Two-way ANOVA of toxin and time revealed no significant interaction (F_(3,32)_ = 0.60, *p *= 0.618, n = 5 per group) of TH expression. There was a significant main effect of toxin (F_(1,32)_ = 421.73, *p *< 0.001) on the optical density of TH positive fibres in the striatum between 6-OHDA and ascorbic acid injected animals (Fig. 2a and b). However, there was no significant main effect of time after 6-OHDA injection (F_(3,32)_ = 0.39, *p *= 0.755), suggesting that the loss of TH positive fibres in the striatum was fully established after just 1 week and didn’t increase any further over time. Similarly, two-way ANOVA of toxin and time resulted in no significant interaction (F_(3,32)_ = 0.40, *p *= 0.753, n = 5 per group) on DAT expression. There was a significant main effect of toxin (F_(1,32)_ = 1554.52, *p *< 0.001) on the optical density of DAT positive fibres in the striatum between 6-OHDA and ascorbic acid injected animals (Fig. 2c, d). However, there was no significant main effect of time after 6-OHDA injection (F_(3,32)_ = 1.27, *p *= 0.299), suggesting the loss of DAT positive fibres in the striatum was fully established after just 1 week and didn’t increase any further over time. The results from this study therefore demonstrate that the modified 6-OHDA model described by Thiele et al. [11] results in significant and progressive dopaminergic and total neuron loss in the SNpc, as well as a loss of TH and DAT positive fibres in the striatum.

### Activin A levels are increased after administration via osmotic micropump

**Fig. 3:**
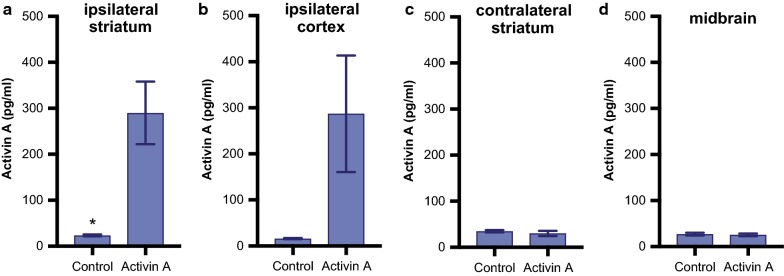
Activin A levels in the striatum, cortex and midbrain, ELISA analysis demonstrates that striatal administration via an osmotic micropump significantly increased levels of activin A in the ipsilateral striatum (**a**) and elevated levels in the ipsilateral cortex (**b**) but not in the contralateral striatum (**c**) or midbrain (**d**) when analysed 2 days later. All values represent the mean ± SEM. * = *p *< 0.05 (n = 5 per group)

Prior to assessing the efficacy of activin A on LID we confirmed that recombinant activin A was diffusing to the target region when administered via an intrastriatal osmotic micropump. Toxin and l-Dopa naive mice received an osmotic micropump containing either activin A or vehicle. Four regions, the ipsilateral striatum, contralateral striatum, midbrain and ipsilateral cortex, were dissected 2 days later. An ELISA was used to quantify activin A levels in these regions. Independent t-tests confirmed significantly increased activin A levels in the ipsilateral striatum (t = 3.907, *p *< 0.05, n = 5 per group) and elevated in the neighbouring cortex (t = 2.144, *p *= 0.099, n = 5 per group). However there was no increase in activin A in either the midbrain (t = 0.418, *p *= 0.687, n = 5 per group) or contralateral striatum (t = 0.695, *p *= 0.507, n = 5 per group), ensuring a region specific diffusion of activin A via striatal micropump (Fig. 3).

### Activin A treatment has no effect on severity or onset of LID

**Fig. 4:**
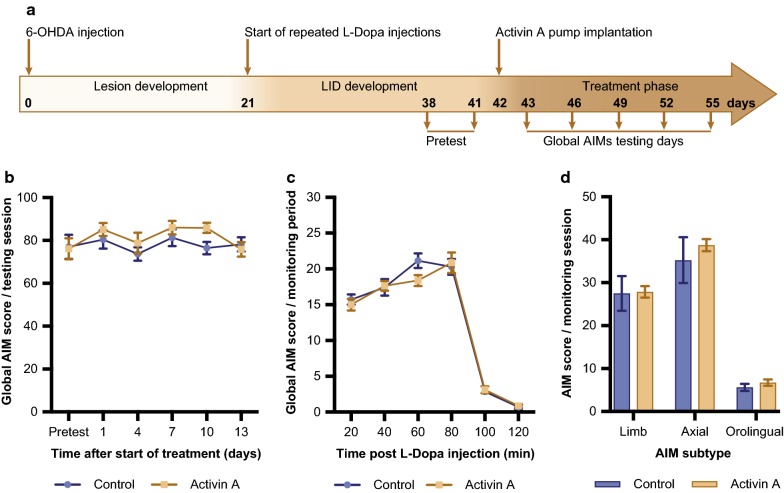
Activin A does not reduce severity of established LIDs, **a** experimental design. Mice are injected with 6-OHDA and develop a lesion over a 3 week period, before receiving repeated l-Dopa injections to establish AIMs during the following 3 weeks. Based on the AIMs score (Pretest) animals are split into 2 even groups and are implanted with an osmotic micropump containing either activin A or vehicle. Daily l-Dopa injections continue and AIMs are scored every 3rd day for 120 min. Activin A did not alter global AIMs score over a testing period of 13 days (**b**), during the monitoring session on the last day (**c**) or as a combination of axial, limb and orolingual scores during the last testing session (**d**). All values represent the mean ± SEM (control n = 7; activin A n = 8)

To test the efficacy of activin A as a treatment to reduce the severity of LID, 6-OHDA lesioned mice were rendered dyskinetic with repeated l-Dopa injections over 3 weeks. Thereafter, mice with LIDs received treatment with vehicle or activin A together with l-Dopa, and abnormal involuntary movements (AIMs) were rated for 13 days (Fig. 4a) to investigate the ability of activin A to reduce the severity of LIDs.

We observed a 16% mortality rate, with 3 out of 19 animals deceasing following 6-OHDA injection. A two-way repeated measures ANOVA revealed no statistically significant effect of activin A on the severity of AIMs (interaction: F_(4,52)_ = 1.978, *p *= 0.112; time F_(4,52)_ = 5.088, *p *< 0.01; treatment: F_(1,13)_ = 1.071, *p *= 0.32, control n = 7, activin A n = 8; Fig. 4b). To investigate a possible effect of activin A on AIMs expression during a single monitoring session, AIM scoring was analysed at each 20 min interval on the final test day (Day 13). Mann–Whitney tests showed no differences between activin A treatment and vehicle at any time point after l-Dopa injections (20 min: U = 27.5, *p *= 0.955; 40 min: U = 25.5, *p *= 0.779; 60 min: U = 12, *p *= 0.072; 80 min: U = 25, *p *= 0.779; 100 min: U = 23.5, *p *= 0.613; 120 min: U = 24, *p *= 0.694; Fig. 4c). Furthermore, Mann–Whitney tests showed no changes in the expression of different subtypes (axial: U = 24, *p *= 0.694; limb: U = 12.5, *p *= 0.072; orolingual: U = 16, *p *= 0.189) between activin A and vehicle (Fig. 4d). Together these results suggest activin A has no effect on reducing severity of established AIMs.

**Fig. 5:**
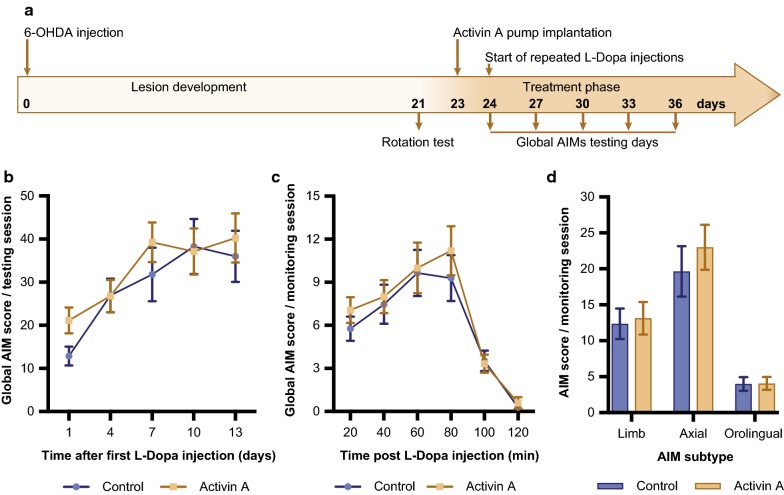
Activin A does not delay the onset of LIDs, **a** experimental design. Mice receive a 6-OHDA injection to develop a lesion over a 3 week period, before undergoing an amphetamine induced rotation test to assess the lesion magnitude. Based on the rotation score animals are split into 2 even groups and receive an osmotic micropump filled with either activin A or vehicle. The following day repeated l-Dopa injections start and AIMs are scored every 3rd day for 120 min. Activin A did not alter global AIMs score over a testing period of 19 days (**b**), during the monitoring session on the last day (**c**) or as a combination of axial, limb and orolingual scores during the last testing session (**d**). All values represent the mean ± SEM (control n = 17; activin A n = 15)

We next aimed to investigate if activin A can delay the onset of LID. Therefore, in a second study, animals were randomized 3 weeks following 6-OHDA injection to receive either daily treatment with l-Dopa + vehicle or l-Dopa + activin A for 13 days (Fig. 5a). In this animal cohort we observed a 19% mortality rate, with 8 out of 42 animals deceased following the 6-OHDA injection. A two-way repeated measures ANOVA revealed no statistically significant effect of activin A on delaying the onset of AIMs (interaction: F_(4,120)_ = 1.387, *p *= 0.2425; time F_(4,120)_ = 24.89, *p *< 0.001; treatment: F_(1,30)_ = 0.3608, *p *= 0.5526, control n = 17, activin A n = 15; Fig. 5b). The significant main effect of time on global AIMs confirmed the previous established LID development profile of approximately 2 weeks [6]. Similar to the above experiments, Mann–Whitney tests on data from the last monitoring session (Day 13) showed no differences between activin A treatment and vehicle at any time point after l-Dopa injections (20 min: U = 99.5, *p *= 0.295; 40 min: U = 117, *p *= 0.710; 60 min: U = 122, *p *= 0.852; 80 min: U = 109, *p *= 0.502; 100 min: U = 127, *p *= 1.0; 120 min: U = 116, *p *= 0.682; Fig. 5c). Furthermore, Mann–Whitney tests on day 13 showed no changes in the expression of different subtypes (axial: U = 102.5, *p* = 0.350; limb: U = 121, *p* = 0.823; orolingual: U = 123.5, *p* = 0.88) between activin A and vehicle (Fig. 5d). Together these results suggest activin A has no effect on delaying the onset of AIMs.

## Discussion

In this study we first confirmed that an updated 6-OHDA mouse model leads to markedly reduced mortality. Furthermore, our analysis of neuron and fibre loss post-6-OHDA administration extends the understanding of the time profile of neurodegeneration in this model. Finally, we are the first to report on the efficacy of activin A as a possible anti-dyskinetic therapy, finding no evidence to indicate it provides benefit, albeit with several important caveats, as discussed below.

### An updated MFB 6-OHDA injection protocol and an enhanced post-operative care paradigm markedly reduces mortality in mice

Assessing the temporal profile of mortality and lesion magnitude following 6-OHDA injection in the MFB of mice provides important reference points for determining the efficacy of potential therapeutics for PD and LID. In the current study 81% of the cohort survived to 21 days, confirming prior reports of markedly improved mortality rates using a modified injection protocol [11]. Thiele et al., hypothesised that a number of factors associated with their modified injection protocol contributed to improved survival rates including altered stereotaxic coordinates, smaller injection volumes, slower infusion rates and reduced cerebral damage through the use of a smaller needle [11]. In our study we adopted this approach and also used a rigorous post-operative care paradigm involving the provision of sugared milk (to stimulate appetite and prevent malnutrition), the use of heating pads throughout the entire protocol (to prevent hypothermia), and daily monitoring and prevention of dehydration, in line with recent recommendations [26]. In animals that did not survive to the endpoint (21 days) we noted an increased prevalence of penile prolapse, a finding previously observed by others [27]. To limit this post-operative complication throughout the study we provided a soft, clean bedding surface and topically applied an antibiotic lubricating jelly, to animals exhibiting signs of prolapse, to reduce further infection and enhance the likelihood of survival [28]. This extensive post-operative care paradigm likely contributed to our enhanced survival rate.

### MFB 6-OHDA injections in mice resulted in peak loss of neuron numbers after 3 weeks

Previous studies have illustrated that a significant SNpc lesion and dopamine deficits in the striatum are observable in mice 3 weeks following MFB 6-OHDA injections, with both required to induce dyskinesia after repeated l-Dopa injections [11]. However, a prolonged analysis of progression of neuron and terminal loss over time using the modified mouse model provided by Thiele et al. has not yet, to the best of our knowledge, been conducted. In the current study we demonstrated that a unilateral 6-OHDA administration into the right MFB resulted in significant and progressive dopaminergic and total neuron loss in the SNpc, which takes 3 weeks to be fully established, before becoming stable thereafter (up to 4 weeks). Another recent study investigated dopaminergic neuron loss in mice with an MFB 6-OHDA lesion during the initial 2 weeks following the 6-OHDA injection [14]. In contrast to our study, the authors observed a more rapid loss of neurons, which may be explained by their use of a higher concentration of 6-OHDA (5 ug, as compared to our 3 ug). Furthermore, they assessed neuron loss by measuring the staining intensity of TH, an enzyme that catalyzes the conversion of the amino acid l-tyrosine to l-Dopa. It has been reported that TH expression can decrease after toxin application [29]. Accordingly, an analysis of TH intensity could reflect a down regulation of the TH enzyme, rather than the loss of neurons. In our study we therefore not only stereologically counted TH positive neurons, but also total neuron numbers in the SNpc using the neuronal marker NeuN. Considering we observed similar patterns in the counts for TH positive neuron numbers and NeuN positive neuron numbers, we are confident our results reflect neurodegeneration.

Administration of 6-OHDA into the MFB of mice is suited for LID studies due to a consistent development of AIMs using this injection location. A range of other 6-OHDA injection sites are however commonly used for PD research. The most common injection site of 6-OHDA in mice is intrastriatal, which produces a slow retrograde degeneration of nigrostriatal neurons over 4–8 weeks and is an ideal model to study neuroprotective treatments for PD [30]. Furthermore, 6-OHDA injections directly into the SN result in a similarly rapid, but less severe lesion, when compared to MFB injected animals, however a detailed time course of lesion development has not yet been reported [10, 31, 32].

### MFB 6-OHDA injections in mice resulted in peak loss of striatal terminals after 1 week

Alongside neuron loss in the SNpc, 6-OHDA injection into the MFB also results in a loss of TH and DAT positive fibres in the striatum. In our study the loss of TH and DAT terminals is already fully established 1 week after 6-OHDA surgery with no further progression over time. Similar studies in rats and mice with MFB lesion are in accordance with our results and depending on the time point of tissue sampling show extensive terminal loss as early as 72 h post 6-OHDA injection [14, 33–35]. The observation of terminal loss in the striatum preceding cell body degeneration in the SNpc is consistent with data from other research groups and is thought to underlie terminals being more sensitive to 6-OHDA toxicity than cell bodies [36–38].

With the results obtained from the present study we conclude that this modified protocol is indeed an improvement over earlier protocols, in that it results in the same magnitude of neurodegeneration within a similar timeframe, whilst markedly reducing mortality. We also validated that the use of a 3 week MFB lesion paradigm ensures a complete lesion of the nigrostriatal tract.

### Exogenous application of activin A has no beneficial effect on the timing of LID onset, or on LID severity

Dopamine replacement is the gold standard therapy for relieving the symptoms of PD-related movement disabilities, despite the development of LIDs within a few years after the initiation of l-Dopa treatment. Traditional therapeutic investigations of LIDs have focused predominantly on pharmacologically targeting dopaminergic or glutaminergic signalling pathways, whilst more recent studies have suggested neuroinflammation as a possible therapeutic target [39]. As part of these efforts, correlative links have been established between the reduction of glial responses and reduced AIMs expression in rodent models of LID [18, 19]. Considering anti-inflammatory therapies are a novel direction for LID research, there are many putative anti-inflammatories that can be screened for efficacy in preclinical models.

In the present study we aimed to extend our prior work assessing the efficacy of the putative neuroprotective and anti-inflammatory molecule activin A in neurodegenerative diseases. In contrast to our prior therapeutic successes with activin A in mouse models of PD [21, 22], we found no evidence in the present study that exogenously administered activin A has anti-dyskinetic capabilities. Specifically, we found no reduction in the severity of AIMs in animals with established dyskinesia, nor was there an effect on the priming of LID. These findings were consistent across every measured time point. Moreover, the time profile in each monitoring session and the combination of subtypes did not differ between PBS and activin A treated animals.

Our study had several strengths, supporting the robustness of the findings. The results of the AIMs assessment were consistent with previous reports [6, 27], mitigating the possibility our results were negative due to any technical errors with our AIMs analysis. Furthermore, we confirmed activin A was reaching our target region (the striatum), removing the possibility activin A had no efficacy due to a lack of diffusion to the striatum.

Our study also has several limitations. First, we only included male mice and thus cannot rule out potential efficacy in female cohorts. Furthermore, we had initially predicted that a potential efficacy of activin A on LIDs may come about via an anti-inflammatory effect, as we have previously shown an anti-inflammatory and neuroprotective effect of activin A in mouse models of PD [21, 22]. Considering we were unable to detect a beneficial effect of activin A on LID, we chose not to initiate an investigation of its anti-inflammatory properties in the current study. We are therefore unable to hypothesise the lack of efficacy was due to some failure, or partial failure, of its putative anti-inflammatory capabilities. We did however demonstrate in the present study that the infused activin A was present in the brain at concentrations previously shown by us to exert beneficial effects [21]. Additionally, although we hypothesised neuroinflammation plays a major role in the development of LIDs, we recognize a causal link between the two has not yet been firmly established.

It is possible therefore that activin A failed to be anti-dyskinetic because neuroinflammation does not play a major role in the development and progression of LID. However, we perceive this as unlikely, considering the growing body of evidence supporting the hypothesis that inflammation does play a role in the development of LIDs [15]. There is conclusive evidence of enhanced neuroinflammation following 6-OHDA injection into the MFB, resulting in increased microgliosis and astrogliosis in the striatum [40]. While some research suggests the most significant changes in microglia and astrocyte activation occurs within a few days of the 6-OHDA injections and that there is a progressive reduction over time [14], other studies show prolonged activated states [41, 42], especially in astrocytes [35]. Furthermore, LID specific research has indicated an increased activation of microglia and release of TNFα in the striatum of dyskinetic mice after repeated l-Dopa injections [15], strengthening the hypothesis of a neuroinflammatory milieu in the striatum of dyskinetic mice. Finally, we note the growing literature that amantadine (the only anti-dyskinetic treatment available in the clinic) may partially provide benefit by inhibiting the inflammatory activation of microglia, despite long being thought to primarily exert its effects through weak NMDA receptor antagonism [43].

## Conclusion

In conclusion this is the first study to investigate exogenous activin A as a therapeutic treatment in animals with LIDs. Although activin A failed to show an effect in reducing the severity or the onset of LID in this study, further research is required to elucidate if activin A is in fact not anti-inflammatory in the striatum, or if any potential anti-inflammatory effect is not sufficient to be anti-dyskinetic. Furthermore, we have established a time course of neuronal loss in the SNpc with subsequent loss of TH and DAT fibres in the striatum using an improved protocol of 6-OHDA injections into the MFB of mice.

## Methods

### Animals

Male C57BL/6 J mice (11 weeks old) were purchased from Australian BioResources (Mona Vale Australia). All animals were acclimatized for 1 week in housing of 4–5 animals per cage, with free access to food and water, and in rooms maintained on a 12-h light/dark cycle. At the commencement of the study animals were housed individually and all experiments were performed during light hours. All surgeries were performed under a ketamine (8.7 mg/ml; Mavlab, Australia) and xylazil (2 mg/ml; Troy Laboratories Pty Ltd, Australia) anaesthesia protocol. Tissue collection for all immunohistochemical experiments was obtained via cardiac perfusion under ketamine/xylazil anaesthesia and all tissue collection for ELISA experiments was obtained via cervical dislocation under isofluorane anaesthesia. Experiments were carried out in accordance with the National Health and Medical Research Council (Australia) guidelines for animal research and were approved by the Garvan Institute and St. Vincent’s Hospital Animal Ethics Committee under approval numbers 12/36 and 15/38.

### Unilateral medial forebrain bundle (MFB) lesioning

Thirty minutes prior to surgery desipramine hydrochloride (Sigma Aldrich, Australia) was administered at 10 ml/kg by intra-peritoneal (i.p.) injection. Mice were then anaesthetized and positioned in a stereotaxic apparatus (Kopf Instruments, USA). Mice received a 0.2 μl injection of 15 mg/ml (total 3 μg) 6-hydroxydopamine hydrobromide (Sigma Aldrich, Australia) in 0.02% ascorbic acid in the right MFB at the following coordinates: AP − 1.2, ML − 1.1, DV − 5.0, relative to bregma and the dural surface, as previously described [11]. The injection rate of 6-OHDA (or 0.02% ascorbic acid control) was 0.1 μl/min and the syringe was left in place for an additional 5 min post injection to allow for complete diffusion into the target area. The incision was sutured (Dynek, Australia) and animals were placed in individual cages on heating pads. During post-operative recovery, mice were provided with recovery gels and sugared milk to ensure adequate nutrition and hydration. One half of the cage was kept on heading pads for the entire study, to allow mice to choose their environment and to preclude hypothermia. Mice were monitored daily for 3 weeks following surgery and were injected subcutaneously with 300 μl glucose (5%) and 300 μl saline (0.9%) [26] if signs of dehydration and malnutrition were present.

### Amphetamine induced rotation test

In order to evaluate the efficacy of the lesion, mice were assessed for amphetamine induced rotations 3 weeks post lesion. Animals received an amphetamine injection (2.5 mg/kg; Sigma Aldrich, Australia, in saline i.p.) and were immediately placed in a clear circular cylinder (diameter 15 cm). After a habituation period of 10 min, ipsilateral and contralateral rotations were recorded for 40 min. Total rotational activity was scored during video analyses. Each 360° rotation was scored, with ipsilateral turns scored as + 1 and contralateral turns as - 1. Results were expressed as ipsilateral net rotation per minute. Animals with a net score of less than 2 rotation per minute were excluded from the remainder of the study.

### Osmotic micro-pump implantation

Deeply anesthetised mice were placed in a stereotaxic apparatus. Osmotic micro-pumps (Alzet, USA) for dyskinesia studies (Model 1002) or for activin A availability studies (Model 1003D) were filled with either 49 ng/μl or 12.25 ng/μl respectively, for a 294 ng total daily dose of recombinant human/mouse/rat activin A (R&D Systems, USA) or 1 × phosphate buffered saline (PBS) control. Pumps were implanted subcutaneously at the back of the neck and an infusion cannula (PlasticsOne, USA) was attached to the micro-pump to deliver activin A to the right striatum at AP + 0.5, ML − 2.2, DV − 3.5 relative to bregma.

### Abnormal involuntary movements (AIMs)

Mice were co-administered l-Dopa methyl ester (6 mg/kg; Sigma Aldrich, Australia, in saline i.p.) and benserazide-HCl (12.5 mg/kg; Sigma Aldrich, Australia, in saline i.p.) to induce AIMs as described below in the experimental design section. Mice were placed individually in transparent plastic cylinders (diameter 15 cm) without bedding material and scored for 1 min every 20th min during the 120 min following l-Dopa administration. Scoring was performed by an investigator blinded to the treatment groups. AIMs were evaluated according to the mouse dyskinesia scale described in detail previously [6, 44]. The AIMs were classified into three subtypes according to their topographic distribution. Axial AIMs are characterized by twisting motions of the neck and upper trunk towards the contralateral side of the lesion. Limb AIMs are rapid uncontrolled movements or dystonic posturing of the contralateral forelimb and orolingual AIMs are movements affecting orofacial muscles and contralateral tongue protrusion. AIMs were scored on 2 different parameters simultaneously on a scale of 1–4 (with 4 being the highest) based on the severity (amplitude scale) and the amount of time they were present (basic scale). A total AIMs score was then produced by multiplying basic score and amplitude score for each AIM subtype, at each monitoring period, and the sum of these scores is referred to as “global AIMs”.

### Experimental design

#### Effects of activin A treatment on severity of LID

Beginning 3 weeks following 6-OHDA lesion surgery animals received repeated l-Dopa and benserazide injections over a 3 week period. During the final week AIMs were scored on 2 occasions (Pretest). Animals that failed to develop AIMs were excluded from the study. Based on the scores of the 2 testing sessions animals were equally divided into 2 treatment groups. On the day after the last pretest animals received an osmotic micropump filled with either activin A or sterile PBS (control). Beginning the day after pump implantation animals received daily l-Dopa and benserazide injections and AIMs expression was measured every 3rd day for a total of 5 testing sessions.

#### Effects of activin A treatment on onset of LID

Amphetamine induced rotations have been shown to correlate with the extent of 6-OHDA lesion, with more rotations indicating increased dopamine depletion [45]. Therefore, 2 days following the amphetamine induced rotation test animals were equally divided into 2 treatment groups, one of which received a micropump filled with activin A, the other receiving sterile PBS (control). On the day after the pump implantation animals received daily i.p. injections of l-Dopa and benserazide and AIM were assessed every 3rd day to obtain a total of 5 testing sessions.

#### Immunohistochemistry

Anaesthetized mice were perfused transcardially 1, 2, 3, or 4 weeks following 6-OHDA injection using ice-cold PBS, followed by 4% paraformaldehyde. Whole brains were collected and processed as previously described in detail [22]. The following primary and secondary antibodies were used in this study: monoclonal mouse tyrosine hydroxylase (TH 1:1000, Sigma Aldrich, Australia, cat # T2928), polyclonal rabbit anti-TH (1:1000, Merck Millipore, Australia, cat # AB152), monoclonal mouse neuronal nuclei (NeuN 1:500, Merck Millipore, Australia, cat # MAB377), monoclonal rat dopamine transporter (DAT 1:1000, Merck Millipore, Australia, cat # MAB369), biotin-labeled antibodies (1:250, Abcam, Australia, cat # AB6813; ThermoFisher Scientific, Australia, cat # B-2770). Immunolabeling was detected with 3,3′-Diaminobenzidine (DAB, Abacus) or Nova-Red (Abacus) according to manufacturer’s instructions.

#### Stereology

SNpc cell populations were quantified using the optical fractionator method and Stereo Investigator 7 software (MBF Bioscience, USA), as previously described [22]. The investigator was blinded to experimental groups during stereological counting.

#### Densitometry

TH-positive and DAT-positive fibres in the striatum were analysed using optical densitometry at the following coordinates relative to bregma: − 0.24 mm, − 0.48 mm, − 0.72 mm, and − 0.96 mm, using Image Pro Plus 6.0 software (Media Cybernetics, USA) as previously described [22].

#### Activin A ELISA

Toxin and l-Dopa naïve mice were sacrificed by cervical dislocation 2 days following activin A pump implantation and the ipsilateral striatum, contralateral striatum, midbrain (substantia nigra + ventral tegmental area) and ipsilateral cortex were rapidly dissected. Tissue homogenates and protein quantification were performed as previously described [21]. Activin A levels were measured via an ELISA (Quantikine Activin A assay, R&D systems, USA) according to manufacturer’s instructions. All samples were analysed in duplicate on the same ELISA plate and the average intra-assay coefficient of variation (CV) was 7.9%.

#### Statistics

All statistical analyses were performed using IBM SPSS Statistics version 25 (SPSS Inc., USA). Shapiro–Wilk tests were performed on all data sets to assess normality, before analysing data either with parametric or non-parametric tests. All data included in the time course of lesion development was assessed by two- way ANOVA followed by Bonferroni post hoc analysis. Data obtained from LID studies were analysed with two- way repeated measures ANOVA or Mann–Whitney tests, while data acquired with activin A ELISA were analysed using independent t-tests (Additional file 1).

## Additional file

**Additional file 1: Datasets.** Raw data used for Figs. 1, 2, 3, 4 and 5.

## Abbreviations

6-OHDA: 6-hydroxydopamine
AIM: abnormal involuntary movement
CV: coefficient of variation
LID: l-Dopa induced dyskinesia
DAT: dopamine transporter
MFB: medial forebrain bundle
NeuN: neuronal nuclei
PBS: phosphate buffered saline
PD: Parkinson’s disease
SEM: standard error of the mean
SNpc: substantia nigra pars compacta
TH: tyrosine hydrolase
